# Rolandic Epilepsy – A Silent Enemy. An Instructive Case

**DOI:** 10.7759/cureus.16741

**Published:** 2021-07-29

**Authors:** Rony Cohen, Avinoam Shuper

**Affiliations:** 1 Neurology, Schneider Children's Medical Center in Israel, Petah Tikva, ISR

**Keywords:** rolandic epilepsy, autism, learning disability

## Abstract

A child is described who was followed in our clinic due to attention deficit hyperactivity disorder (ADHD) and was found to have the following list of diagnoses: mild developmental delay, motor tics, learning disability, selective mutism and autistic-like features. These disorders became manifest and were diagnosed over a period of several years in the above-noted order. He never had seizures. Medical evaluation, which was initiated due to his learning disability, was negative. The last test which was done was EEG, and this revealed a very active picture compatible with Rolandic epilepsy. Eventually, this was the key for the understanding of his whole clinical picture. It is suggested that in children with learning disability, the possibility of having seizure-free Rolandic epilepsy be considered early in the course of evaluation.

## Introduction

Learning disabilities are frequent disorders that prompt consultation with primary paediatricians and referrals to paediatric neurologists. Speech and language delays in children are associated with increased difficulty with reading, writing, attention and socialization. Children with learning disabilities have been found to have neurophysiological dysfunction, manifested by atypical neurophysiological activation patterns in brain areas involved with the control of attention, executive functions, planning and language functions [1]. Generally, electroencephalogram (EEG) is not recommended in the routine medical evaluation of children with learning disabilities, unless they have seizures. We describe a potential role of EEG in the evaluation of children with learning disabilities but without seizures.

## Case presentation

**Figure 1:**
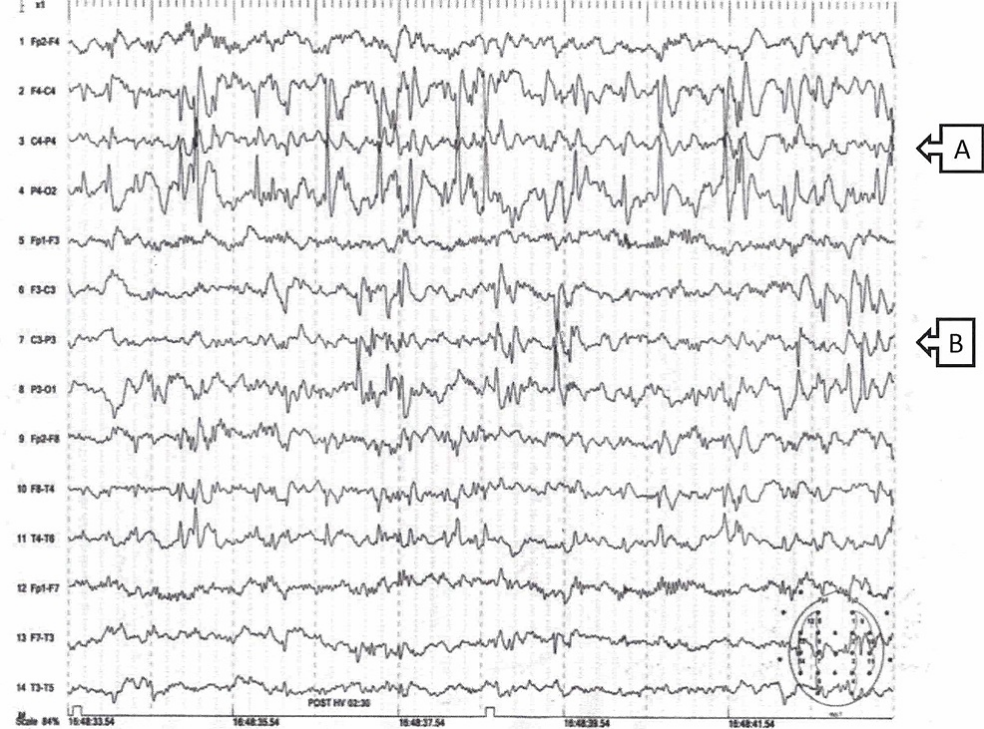
Sleep-deprived EEG recording of the patient. Centro-temporal phase-reversal (A) on the right side and (B) on the left.

A seven-and-a-half-year-old boy presented to the neurology clinic for evaluation of a presumed diagnosis of attention deficit hyperactivity disorder (ADHD). His history included a normal pregnancy and delivery, and good health during early childhood. Due to short attention span and poor school achievements, he was diagnosed as having a marked learning disability and ADHD, and he was referred to a special education class. His physical examination was normal except for hyperactivity, and the impression of difficulty with eye contact. He had mild motor tics disorder. Developmental evaluation revealed borderline intelligence with an IQ of 76%. During follow-up evaluation at age 9 years, the mother reported that at specific situations the child turns speechless. These events were noted to occur mainly when the child was asked to communicate with strangers. When this happened, the child did not lose consciousness, but would not respond verbally. He did not have any automatisms during this time. The diagnosis of selective mutism was made and confirmed by psychiatric evaluation. The boy experienced marked reading difficulties; his reading ability was at the first-grade level. He was referred for a sleep and awake EEG, which showed very frequent unrelated epileptiform activity over the parieto-occipital region on the right side and the parieto-temporal on the left side, in the form of a triphasic spike-wave pattern. The EEG was compatible with Rolandic epilepsy (Figure 1).

The child had never had seizures. The EEG results were discussed with the parents. They were against medical treatment.

## Discussion

The diagnoses of the presented child included mild developmental delay, ADHD, motor tics disorder, learning disability, selective mutism, autistic-like features and very active Rolandic type EEG without seizures.

This child is of interest, because despite the absence of seizures, the EEG, which was performed without a clear indication, contributed to understanding his full clinical picture. Indeed, his list of symptoms characterizes children with Rolandic epilepsy.

Wirrell et al. [2] described benign Rolandic epilepsy as occurring “in neurologically and cognitively normal children who present with a nocturnal partial seizure. The seizure often begins focally with clonic movements of the mouth, increased salivation, and guttural sounds, and progresses to secondary generalization. Dysarthria, stiffness of the tongue and jaw, and a choking sensation are frequently described. The EEG shows dramatic, high-voltage, centrotemporal spikes, often followed by slow waves that are activated by sleep and often shift from side to side.”

Of Wirrell et al.’s cohort of 42 children with atypical Rolandic symptomatology, 8 (29%) had developmental delay and 2 (5%) had ADHD or Tourette syndrome. Three (7.1%) had abnormal background EEG. However, as ADHD can present in siblings unaffected by Rolandic epilepsy, it was suggested that attentional difficulties are not explained by the presence of the seizures or spikes associated with Rolandic epilepsy; rather, their presence may be due to susceptibility factors shared between attention deficit disorders and Rolandic epilepsy.

The selective mutism in our patient is of interest. Politi et al. [3] described six children with selective mutism, two of them had a similar EEG pattern as our patient. In one of the patients, the mutism resolved. In the present patient, some improvement was found as well.

Wergeland in 1979 [4] wrote as follows: “The term elective mutism (synonymous to selective mutism) was first used by Tramer in 1934 to differentiate between total mutism and mutism that is limited to the absence of verbal communication with certain people. The latter implies that the child has both learned a language and is capable of using it. There is consequently no speech defect but a disturbance of communication. Other forms of communication, such as mimic or facial expression and physical forms of expression, are intact. Generally, the child speaks naturally with one or several family members, but is completely mute with others. The mutism may be very occasional, and present in relation to the family or when speaking to others outside the home.”[4] For a minority of children with selective mutism, delays in speech development or difficulty in articulation are reported.

In a cohort of children with selective mutism, the mean age for onset of symptoms was 4.5 years and the mean age at diagnosis was 8.8 years. Autism spectrum disorder was diagnosed in 63% of the study group, with no gender difference. Those with both selective mutism and autism spectrum disorder were characterized by later onset of symptoms and older age at diagnosis; a history of speech delay, and borderline IQ or intellectual disability were more common. The authors concluded that the results highlight the risk of overlap between autism spectrum disorder and selective mutism [5].

Already at an early age, our patient showed difficulties with eye contact. Psychiatric evaluation ruled out the diagnosis of autism. Up to one-third of children with autism have epilepsy, and epileptiform EEGs are not uncommon, even in those without a history of clinical seizures. Although regression occurs equally in children with and without epilepsy, it is more prevalent in those with epileptiform EEGs [6]. Some patients, especially those in whom the epileptic process is localized around the perisylvian cortex, present with features of autistic spectrum disorder, but unlike primary autism, there is no loss of social interaction [7].

Cognitive and behavioural comorbidities are often seen in children with epilepsy. In epileptic encephalopathies, frequent seizures and interictal epileptiform abnormalities exacerbate neurocognitive dysfunction, owing to synaptic reorganization or impaired neurogenesis, or to other effects on developing neural circuits. These prompt initiation of effective antiepileptic therapy to limit cognitive comorbidities. Notably, some have claimed that epilepsy, and not EEG abnormalities, is associated with regression in autism and in impaired mental functioning in childhood [8]. Presumably, the current report demonstrates regression with EEG abnormalities alone. Without having seizures, this child may not be diagnosed as having epilepsy.

Overall, our patient has a progressive disorder that was first manifested with mild developmental delay and ADHD, and that progressed to selective mutism. At follow-up, his clinical presentation included electroencephalographic pattern of Rolandic epilepsy, with its diverse symptoms.

Ross et al. [9] reported the progression of symptomatology in Rolandic epilepsy and differentiated between the premorbid and post morbid presentations. At initial presentation, 28.3% of the children had a neuropsychiatric concern. At the postmorbid time, 34/5% reported a neuropsychiatric diagnosis. Nevertheless, 9 years after their study enrollment, the mean full-scale IQ was 106.9. That means that the progressive neuropsychiatric morbidity, eventually resolves as well.

Our case shows that the progression of the psychiatric symptoms can be unrelated to the appearance of seizures, as he never had seizures. Thus, at least in such cases, one cannot distinguish between pre- and post-morbid presentations if they are defined according to the presence of seizures. It seems that CECTS is a continuum of symptomatology which is unrelated to the presence of epilepsy.

The intrinsic disturbance described was manifested on one hand by an abnormal electrographic disorder, and on the other hand by progressive developmental regression. This may be considered a maturational disorder, which was evident by the clinical progression, without any change in the child's daily life. The latter may be the reason the parents refused treatment.

Learning point

The basic dysfunction that is included under the umbrella of Rolandic epilepsy may manifest with electrographic abnormality, and not necessarily include overt seizures. The reported case represents progressive dysfunction in a child due to a broad spectrum of clinical manifestations all associated with the "Rolandic epilepsy trait", albeit without overt seizures. We suggest that sleep EEG should be considered for children with learning disabilities, especially in complicated cases, as early as the suspicion of morbidity becomes evident, even without seizures.

## Conclusions

The basic dysfunction that is included under the "umbrella" of Rolandic epilepsy may manifest with electrographic abnormality, and not necessarily include overt seizures. Thus, it is questionable whether similar children may be diagnosed as epileptic. The reported case represents progressive dysfunction in a child due to a broad spectrum of clinical manifestations all associated with the "Rolandic epilepsy trait", albeit without overt seizures. We suggest that sleep EEG should be considered for children with learning disabilities, especially in complicated cases, as early as the suspicion of morbidity becomes evident, even without seizures.
